# Colistin Induces *S. aureus* Susceptibility to Bacitracin

**DOI:** 10.3389/fmicb.2018.02805

**Published:** 2018-11-20

**Authors:** Wei Si, Liangliang Wang, Valentine Usongo, Xin Zhao

**Affiliations:** ^1^Department of Animal Science, McGill University, Montreal, QC, Canada; ^2^College of Animal Science and Technology, Northwest A&F University, Yangling, China; ^3^School of Pharmaceutical Sciences, Tsinghua University, Beijing, China

**Keywords:** bacitracin, colistin, *Staphylococcus aureu*s, antibiotic, susceptibility

## Abstract

Bacitracin has been used in topical preparations with polymyxin B for bacterial infections. Colistin belongs to the polymyxin group of antibiotics and is effective against most Gram-negative bacilli. This study investigated whether colistin could affect the susceptibility of *S. aureus* to bacitracin. *S. aureus* isolates were first incubated with colistin and the susceptibility of *S. aureus* to bacitracin was increased. The effect of the combination of colistin and bacitracin on *S. aureus* was then confirmed by the checkerboard assay and the time-kill kinetics. The Triton X-100-induced autolysis was significantly increased after *S. aureus* was exposed to colistin. Exposure to colistin also led to a less positive charge on the cell surface and a significant leakage of Na^+^, Mg^2^, K^+^, Ca^2+^, Mn^2+^, Cu^2+^, and Zn^2+^. Finally, disruptions on the cell surface and an irregular morphology were observed when the bacteria were exposed to colistin and bacitracin. Bacitracin had a stronger antibacterial activity against *S. aureus* in the presence of colistin. This could be due to the fact that colistin damaged the bacterial membrane. This study suggests that combination of colistin with bacitracin has a potential for treating clinical *S. aureus* infections.

## Introduction

*Staphylococcus aureus* is part of the commensal microorganisms in humans and approximately 20% of healthy individuals are carriers (Gordon and Lowy, 2008). Although *S. aureus* is not always pathogenic, it is a major cause of a wide range of human diseases, especially skin and soft-tissue infections. *S. aureus* infections are increasingly difficult to eradicate due to the emergence of antimicrobial resistance. In fact, infections by *S. aureus* were well controlled by methicillin up until the emergence of methicillin resistance *S. aureus* (Hiramatsu et al., 2001). Although vancomycin has been the most reliable and effective therapeutic agent against MRSA infections, an alarming emergence of vancomycin-resistant *S. aureus* strains have recently been reported in many countries such as in Iran, Europe, United States (Limbago et al., 2014; Askari et al., 2015; Friães et al., 2015). At the same time, the discovery and development of novel and efficient antibiotics has been significantly decreased. In the battle against rapidly emerging bacterial resistance, rational approaches to use existing antibiotics could be more efficient than discovery of new antibiotics.

Bacitracin is a polypeptide antibiotic produced by stains of *Bacillus licheniformis* and *Bacillus subtilis* and has been clinically used for staphylococcal (such as *S. aureus*) infections since the late 1940s (Johnson et al., 1945). Bacitracin functions as an inhibitor of cell wall biosynthesis through its binding to the undecaprenyl pyrophosphate (Stone and Strominger, 1971). The combination of bacitracin with other antibiotics has been efficient to be used as a topical agent (Werner and Russell, 1999). In the United States, ophthalmic ointments containing bacitracin, polymyxin B and neomycin have been shown to eradicate coagulase-negative staphylococci (CNS) from the human skin surface and the underlying stratum corneum as well as preventing repopulation with resident flora (Hendley and Ashe, 1991). Whether other polymyxin has similar effects like polymyxin B is unknown.

Colistin is one of the polymyxins, known as polymyxin E. Colistin is effective against most Gram-negative bacilli, and especially effective to treat infections caused by multi-drug-resistant Gram-negative bacteria (MDR-GNB) owing to its favorable properties of rapid bacterial killing, and an associated slow development of resistance (Li et al., 2006). Colistin is able to bind lipopolysaccharide (LPS) (Warren et al., 1985). Colistin kills Gram-negative bacteria mainly through disruption of outer membrane integrity of bacteria resulting from binding of the antibiotic with LPS and displacing of divalent cations (Yu et al., 2015).

Several studies have claimed that the combination therapy of colistin and other antibiotics are more efficient than a single antibiotic therapy to treat serious infections. For example, Souli et al. (2009) reported that combination of colistin and imipenem was synergistic (50% of isolates) or indifferent (50%) against colistin-susceptible blaVIM-1-type metallo-β-lactamase-producing (MBL) *K. pneumoniae* strains, while it was antagonistic (55.6%) and rarely synergistic (11%) against colistin-resistant strains. In another study, sub-inhibitory concentrations of meropenem (0.06–8 mg/L) and colistin (0.12–1 mg/L) were synergistic against 13 out of 51 *P. aeruginosa* isolates at 24 h, while the combination of meropenem (0.03–64 mg/L) and colistin (0.06–8 mg/L) showed synergy against *A. baumannii* isolates (Pankuch et al., 2008). Song et al. (2008) evaluated the effectiveness of the combination of CMS (colistin methanesulfonate) and rifampicin in 10 patients with ventilator-associated pneumonia caused by carbapenem-resistant (only susceptible to colistin) *A. baumannii* strains and found that seven out of 10 patients benefited from the combination therapy (Song et al., 2008). Recently, it has been shown that colistin potentiated the activity of drugs such as isoniazid and amikacin *in vitro* against *Mycobacterium tuberculosis* (Bax et al., 2015). Owing to its increasing use and reported efficacy in combination therapy against a variety of bacterial infections, in this study we wanted to investigate whether the antibacterial effect of bacitracin against *S. aureus* could be enhanced by colistin.

## Materials and Methods

### Bacteria Strains, Growth Conditions, and Resistance Detection

*S. aureus* BA01611 (MRSA, Wu et al., 2015), *S. aureus* BA01511 (from our laboratory), *S. aureus* WS1 (Li et al., 2015), *S. aureus* RN4220, *S. aureus* Mu50 (MRSA), *S. aureus* ATCC 29213 and *S. aureus* Newman were used in this study. The Mueller-Hinton (MH) medium was used for the growth of *S. aureus*. MICs for antimicrobial agents were determined by using an agar dilution method recommended by the Clinical and Laboratory Standards Institute (2015). The MIC was determined as the lowest concentration at which growth was completely inhibited. Determination of MICs was repeated three times.

### Susceptibility Screen

The susceptibility screen assay was performed as described previously (Haaber et al., 2015). *S. aureus* strains were grown overnight and the cultures were adjusted to subsamples of 5 × 10^5^ CFU/mL in the warm MH broth. Colistin sodium sulfate (Sigma) was added as an inducer at the concentrations of 1/2 MIC (64 μg/mL) for each strain. After 90 min at 37°C with shaking at 180 rpm, 10 μL aliquots of the cultures were spotted on MH agar plates containing bacitracin (Sigma) at a concentration of 1/2 MIC (64 μg/mL). The plates were incubated overnight at 37°C before checking the bacterial growth. Each experiment was repeated three times.

### Determination of the *in vitro* Effects of Combinations of Bacitracin and Colistin

The antimicrobial combination assays were conducted with bacitracin plus colistin by using the broth microdilution checkerboard technique (Mataraci and Dosler, 2012). The test was performed using 96-well microtiter plates containing colistin and bacitracin in twofold serial concentrations. Bacterial suspensions were prepared to yield a final inocula of ∼5 × 10^5^ CFU/mL. Plates were read after overnight incubation at 37°C. Fractional Inhibitory Concentration (FIC) Index was calculated according to the formulas: FIC_bacitracin_ = MIC_bacitracin+colistin_/MIC_bacitracin_, FIC_colistin_ = MIC_bacitracin+colistin_/ MIC_colistin_, FIC Index = FIC_bacitracin_+ FIC_colistin_. FIC Index values were interpreted according to Mun et al. (2013): synergy (FIC Index ≤ 0.5); partial synergy (FIC Index > 0.5 to ≤ 0.75); additivity (FIC Index > 0.75 to ≤ 1); no interaction (indifference) (FIC Index > 1 to ≤ 4) and antagonism (FIC Index > 4.0) (Mun et al., 2013). Each experiment was repeated three times.

### The Time-Kill Assay

The time-killing curve assays were performed in triplicate to study the effect of the combination of bacitracin and colistin on *S. aureus* BA01611 growth as previously described by Mun et al. (2013) with minor modifications (Mun et al., 2013). A single bacterial colony was added to 2 mL of the MHB and grown overnight at 37°C with shaking at 180 rpm. The overnight culture was diluted with pre-warmed MHB to obtain a starting inoculum of approximately 5 × 10^5^ CFU/mL. The *S. aureus* BA01611 strain was exposed to colistin at the concentrations of 0 or 1/2 MIC (64 μg/mL) in the presence or absence of 1/2 MIC (64 μg/mL, except 8 μg/mL for *S. aureus* BA01511) bacitracin. Samples were taken at 0, 2, 4, 6, 8, 16, and 24 h, serially diluted, spread on drug-free plates, and incubated at 37°C for 24 h before counting the colonies. Each experiment was repeated three times.

### Autolysis Assay

The autolysis assay was performed as described previously (Meehl et al., 2007). *S. aureus* BA01611 isolate was grown overnight and diluted in the pre-warmed MH broth and grown to the mid-exponential phase. The cells were exposed to 64 μg/mL colistin while the control was not exposed to colistin. After 1 h of exposure, the cultured cells were washed twice in cold sterile distilled water and re-suspended in the same volume of 0.05 M Tris-HCl, pH 7.2, containing 0.05% Triton X-100. Cells were incubated at 30°C, and the OD_600_ was measured every 30 min. Data are expressed as the percent loss of OD_600_ at the indicated times compared to findings at time zero. Each experiment was repeated three times.

### Determination of Whole-Cell Surface Charges

The determination of whole-cell surface charges was performed as described previously (Meehl et al., 2007). After culturing overnight, *S. aureus* BA01611 was diluted in the pre-warmed MH broth to ensure continued exponential growth during treatment. Cells were treated with and without colistin for 1, 2, and 4 h before harvesting at the mid-exponential phase. After washing twice by centrifugation in 20 mM MOPS buffer, pH 7, the cells were re-suspended in the neutral pH MOPS buffer to a final OD_600_ of 0.6 before adding the positively charged cytochrome c (Sigma) at a concentration of 0.125 mg/mL. The mixture was incubated at room temperature for 10 min before centrifugation and the amount of unbound cationic cytochrome c in the supernatant was quantified photometrically at 530 nm. Each experiment was repeated three times.

### Ion Leakage

The leakage of ions was performed as described previously (Miksusanti et al., 2008). Bacterial cells (*S. aureus* BA01611) were incubated for 24 h and then diluted in pre-warmed MH broth to ensure continued exponential growth during treatment. Cells were exposed to colistin (64 μg/mL) for 1 and 2 h. The bacterial cells without colistin exposure were used as a control. Bacterial cells were harvested by centrifugation at 12,000 × *g* for 15 min and washed twice with cold deionized water. The cells were then adjusted to the same OD_600_ and the dry weight was determined followed by digestion in a HNO_3_/H_2_O_2_ solution at 100°C for 24 h. After dissolution, the cells were centrifuged and the supernatant were recovered and assayed for total mineral content of cells (K^+^, Na^+^, Ca^2+^, Mg^2+^, Zn^2+^, Cu^2+^, and Mn^2+^) by ICP Mass Spectrometers (Varian 810/820-MS). Each experiment was repeated three times.

### Scanning Electron Microscope (SEM)

SEM were performed as described previously (You et al., 2013). *S. aureus* BA01611 cells were treated with 1/2 MIC (64 μg/mL) colistin and/or 1/2 MIC (64 μg/mL, except 8 μg/mL for *S. aureus* BA01511) bacitracin for 1 h and 2 h. Untreated controls were also prepared. The bacterial cells were collected via centrifugation at 10,000 × *g* and then the pellet formed was washed with PBS for three times. Fixation was done by suspending the bacterial cells into 0.25% of glutaraldehyde solution (in PBS, pH 7.0) and then incubated at room temperature for 1 h before collecting the fixed bacterial pellet. Dehydration of the bacterial cells was done by washing the pellets with ethanol at different concentrations up to 100%. After the critical-point drying, the bacterial cells were observed with a field-emission scanning electron microscopy (FE-SEM; FEI Inspect F50).

## Results

### Existence of the Effect of the Combination of Colistin and Bacitracin Against *S. aureus*

**FIGURE 1:**
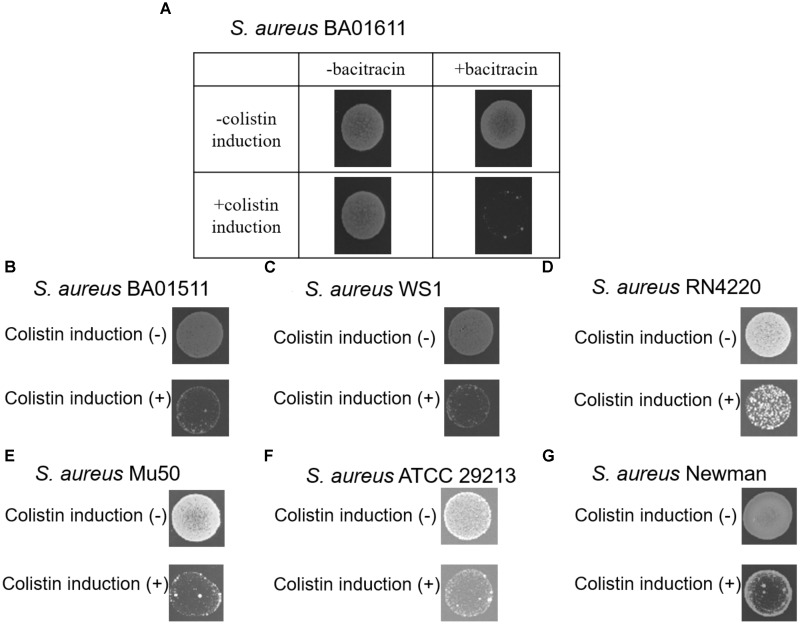
The ability of seven *S. aureus* stains to form colonies at a concentration of 1/2 MIC (64 μg/mL, except 8 μg/mL for *S. aureus* BA01511) bacitracin was assayed after pre-exposure to colistin for 90 min. **(A)** *S. aureus* BA01611 isolate was also incubated on MHA without bacitracin, **(B)** *S. aureus* BA01511, **(C)** *S. aureus* WS1, **(D)** *S. aureus* RN4220, **(E)** *S. aureus* Mu50, **(F)** *S. aureus* ATCC 29213, and **(G)** *S. aureus* Newman.

In order to determine whether colistin would induce *S. aureus* susceptibility to bacitracin, seven *S. aureus* strains were screened. *S. aureus* strains were first treated with colistin at a concentration of 1/2 MIC (64 μg/mL), then 10 μL aliquots of the cultures were spotted on freshly prepared MH agar plates containing bacitracin at a concentration of 1/2 MIC (64 μg/mL, except 8 μg/mL for *S. aureus* BA01511). As shown in Figure 1, such treatments inhibited the growth of all seven strains, suggesting that colistin could induce *S. aureus* susceptibility to bacitracin.

**FIGURE 2:**
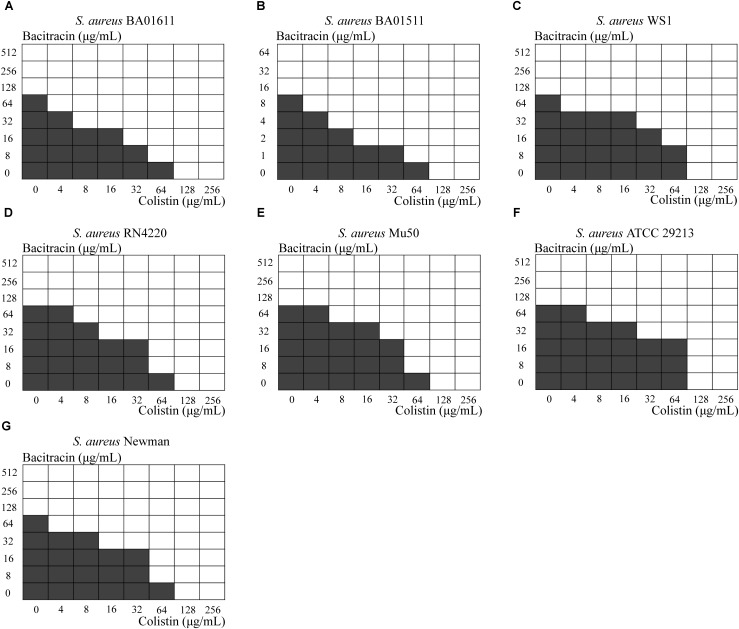
Results from the checkerboard test. Shading indicates visible growth. The Fractional Inhibitory Concentration (FIC) was calculated. **(A)** *S. aureus* BA01611 (FIC index = 0.3125), **(B)** *S. aureus* BA01511 (FIC index = 0.2500), **(C)** *S. aureus* WS1 (FIC index = 0.53125), **(D)** *S. aureus* RN4220 (FIC index = 0.375), **(E)** *S. aureus* Mu50 (FIC index = 0.5000), **(F)** *S. aureus* ATCC 29213 (FIC index = 0.3750) and **(G)** *S. aureus* Newman (FIC index = 0.3750).

Next, a checkerboard assay was performed for each *S. aureus* strain, using several concentrations ranging from below the MIC to 2x MIC. As shown in Figure 2. synergy as indicated by FIC index ≤ 0.5 was obvious in six out of seven strains, while *S. aureus* WS1 showed a partial synergy with the FIC index equal to 0.53125.

**FIGURE 3:**
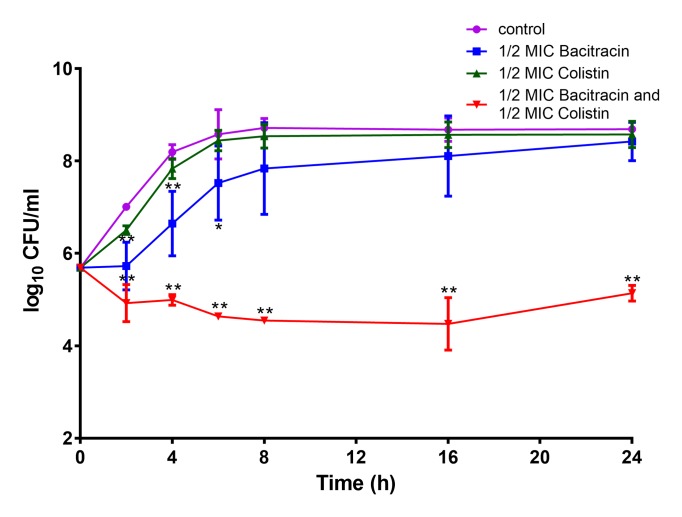
Time-kill curves of colistin and/or bacitracin against *S. aureus* BA01611. Data are presented as means ± standard deviations. The statistical analysis was performed using a one-way analysis of variance (ANOVA) with the least significant difference (LSD) test. ^∗^*P* < 0.05, ^∗∗^*P* < 0.01 in comparison with the control group.

Furthermore, a time-kill analysis was carried out with one MRSA strain *S. aureus* BA01611. As shown in Figure 3, colistin alone or bacitracin alone did not lead to cell growth inhibition after 24 h incubation compared to the control. The combination of colistin and bacitracin resulted in a > 2log_10_ decrease compared to the control after the 24 h incubation period, indicating that the efficiency of the combination of colistin and bacitracin against *S. aureus* strains.

### Autolytic Activity Is Increased in *S. aureus* Treated With Colistin

**FIGURE 4:**
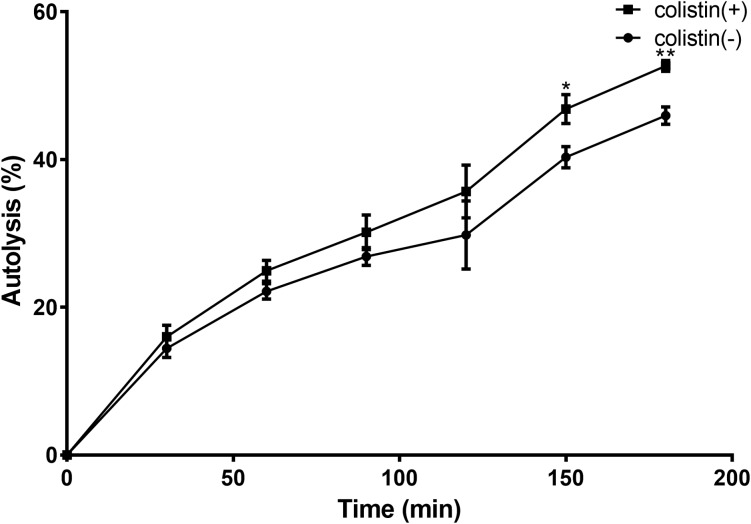
Triton X-100 autolysis assay of *S. aureus* BA01611. Cells were grown with or without colistin for 1 h before being washed and resuspended in buffer containing the detergent. Absorbance at 600 nm was determined for 3 h. Autolysis was determined based on the decrease in the optical density over time relative to that at time zero for each sample. Data are presented as means ± standard deviations. The statistical analysis was performed using a one-way analysis of variance (ANOVA) with the least significant difference (LSD) test. ^∗^*P* < 0.05, ^∗∗^*P* < 0.01 in comparison with the control group.

To investigate how colistin induced *S. aureus* susceptibility to bacitracin, a Triton X-100-induced autolytic activity of the BA01611 strain was first measured after exposure to colistin. As shown in Figure 4, exposure of *S. aureus* BA01611 to colistin at a concentration of 64 μg/mL increased autolysis in comparison with that of the unexposed strain. Triton lysis profiles differed significantly after 150 min exposure to colistin (0.468 ± 0.0196 vs. 0.403 ± 0.0142 after 150 min: 0.527 ± 0.0073 vs. 0.460 ± 0.0117 after 180 min). The results indicated that the susceptibility to bacitracin after colistin induction could be due to the damaged cell structure.

### Whole-Cell Surface Positive Charge Decreased in *S. aureus* Treated With Colistin

**FIGURE 5:**
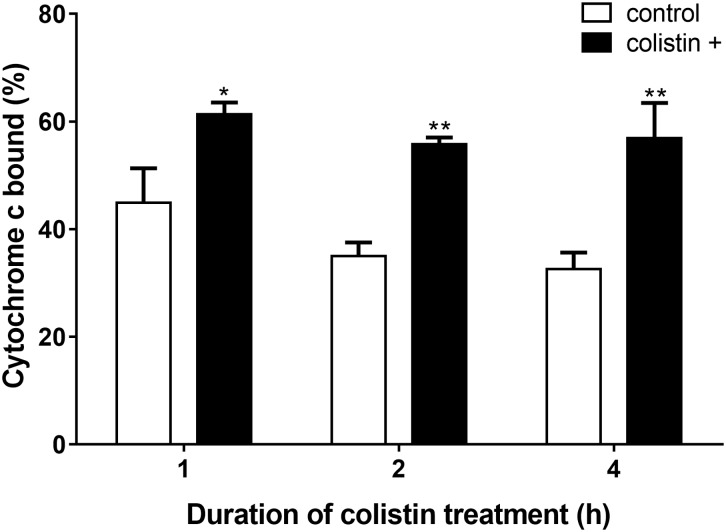
Detection of cationic cytochrome c binding to whole *S. aureus* BA01611 cells in the presence or absence of colistin. The figure shows the percentage of bound cytochrome c after incubating *S. aureus* BA01611 with colistin for 1, 2, or 4 h. Data are presented as means ± standard deviations. The statistical analysis was performed using a one-way analysis of variance (ANOVA) with the least significant difference (LSD) test. ^∗^*P* < 0.05, ^∗∗^*P* < 0.01 in comparison with the control group.

As colistin is known as an important cationic antimicrobial peptide which could displace calcium and magnesium, whether the electrostatic nature of *S. aureus* BA01611 surface was affected by colistin was then determined by adding cationic cytochrome c. As shown in Figure 5, bacterial cells treated with colistin had a significantly greater quantity of bound cytochrome c than non-exposed strains. These results implied that colistin might render the bacterial cell membranes less positively charged and make the bacterial cells more susceptible to other antibiotics.

### Effects of Colistin on Ions Leakage of *S. aureus*

**FIGURE 6:**
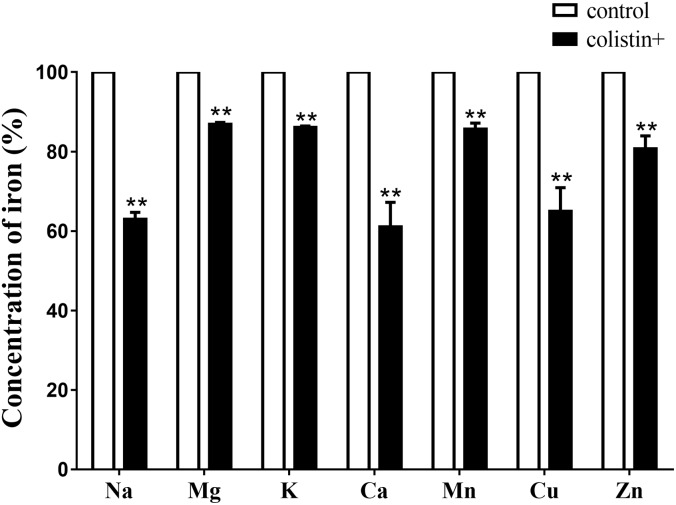
Ion leakages when *S. aureus* BA01611 was exposed to colistin for 2 h. The samples were digested in a HNO_3_/H_2_O_2_ solution of 6 mol/L. The total mineral contents of the bacteria were measured by a Varian 810/820-MS ICP Mass Spectrometer. Data are presented as means ± standard deviations. The statistical analysis was performed using a one-way analysis of variance (ANOVA) with the least significant difference (LSD) test. ^∗^*P* < 0.05, ^∗∗^*P* < 0.01 in comparison with the control group.

To further investigate whether *S. aureus* ions leakage occurred upon colistin exposure, total mineral content of cells (K^+^, Na^+^, Ca^2+^, Mg^2+^, Zn^2+^, Cu^2+^, and Mn^2+^) of *S. aureus* BA01611 was determined by ICP Mass Spectrometers. While exposure to colistin for 1 h did not affect the concentration of the majority of ions, exposure to colistin for 2 h led to significant decrease in concentration of ions: Na^+^ by 63.0%; 86.9% for Mg^2+^; 86.1% for K^+^; 61.1% for Ca^2+^; 85.7% for Mn^2+^; 65.0% for Cu^2+^ and 80.7% for Zn^2+^ (Figure 6).

### Cell Surface Was Damaged When *S. aureus* Co-treated With Colistin and Bacitracin

**FIGURE 7:**
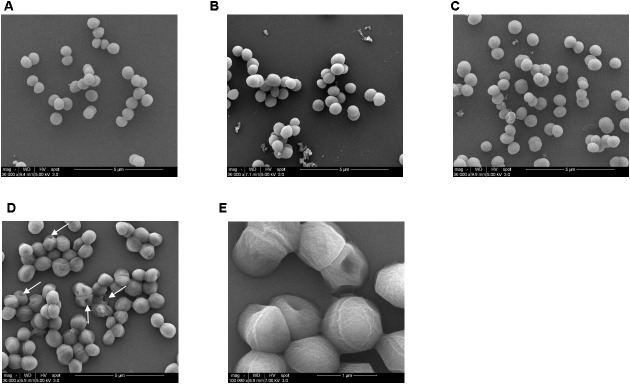
SEM images of *S. aureus* BA01611 cells. **(A)** Untreated *S. aureus* BA01611 (control); **(B)** *S. aureus* BA01611 treated with colistin; **(C)** *S. aureus* BA01611 treated with bacitracin; **(D)** *S. aureus* BA01611 treated with both colistin and bacitracin. Arrows indicated partially damaged surface; **(E)** high magnification of *S. aureus* BA01611 treated with both colistin and bacitracin. The cell surface was damaged in multiple locations.

In order to further confirm that the affected cell surface was involved in the effect of colistin to increase susceptibility to bacitracin, *S. aureus* BA01611 was exposed to 1/2 MIC (64 μg/mL) colistin in the presence or absence of 1/2 MIC (64 μg/mL, except 8 μg/mL for *S. aureus* BA01511) bacitracin for 1 and 2 h and the structure of the cell surfaces was visualized by SEM. As shown in Figure 7, the surfaces of the untreated cells were relatively smooth and continuous with good structural integrity (Figure 7A). With the treatment of colistin or bacitracin, grape-like cell clusters were shown and the cell boundaries were faint and unclear (Figures 7B,C). In the combined treatment of colistin and bacitracin, the morphology of *S. aureus* changed significantly: many ghost cells with irregular morphology, and irregular and distorted outlines of the cell packets. In addition, the cells and clumps were larger than those with or without the treatment of colistin or bacitracin alone (Figures 7D,E).

## Discussion

The rise of antimicrobial resistance in *S. aureus* is a major challenge in both hospitals and community settings (Ventola, 2015). Currently, 60–90% of *S. aureus* infections are reported to be methicillin-resistant (MRSA), and people with MRSA are estimated to be 64% more likely to die than people with a non-resistant form of the infection (WHO, 2014). The limited availability of effective therapies against *S. aureus* has increased the quest to discover new treatment options including the development of new antibiotics as well the combination of antibiotics currently in use in clinical practice. In this study, we found that the antibacterial effects of bacitracin against *S.* *aureus* was enhanced by colistin.

Most studies on combination therapy involving colistin have focused on Gram-negative bacteria. For example, colistin combined with nisin A or pediocin PA-1/AcH synergistically inhibited the enterohemorrhagic bacterium *E. coli* O157:H7 (Naghmouchi et al., 2013). Similarly, several antibiotics such as rifampicin, imipenem, doxycycline, azithromycin, and ciprofloxacin worked synergistically with colistin against *P. aeruginosa* (Biswas et al., 2012). However, Haaber et al. (2015) discovered that susceptibility of *S. aureus* to vancomycin was reduced by concurrent exposure to colistin. Bacitracin is proposed as one of the most popular topical antibiotics and usually combined with polymyxin B in a number of commercially available ointments and creams that are used on minor cuts and abrasions as well as postsurgical wounds (Gette et al., 1992; Spann et al., 2009). Additionally, bacitracin was effective against methicillin-resistant *S. aureus* *in vitro* (Chapnick et al., 1996) and *in vivo* (Soto et al., 1999). Therefore, combination of bacitracin and colistin could be an alternative for the treatment of *S. aureus* infections. This study explored that possibility *in vitro* and our results showed for the first time that colistin enhanced the inhibitory effect of bacitracin against *S. aureus*. Seven *S. aureus* strains used in this study were resistant to colistin with high MIC values. Generally speaking, Gram-positive bacteria are generally less sensitive to polymyxins than Gram-negative bacteria (Storm et al., 1977). The increased susceptibility of *S. aureus* to bacitracin by colistin was confirmed by several assays: the spotted assay, the checkerboard assay and time kill kinetics. Moreover, the damaged and irregular surface of *S. aureus* when co-exposure to colistin and bacitracin confirmed the effectiveness of this combination treatment.

How colistin increased susceptibility of bacitracin against *S. aureus* remains unknown. Because colistin kills Gram-negative bacteria by damaging the bacterial cell membrane and in addition to the synergy observed with other antibiotics against Gram-negative bacteria that has been reportedly linked to this function (Biswas et al., 2012), the effects of colistin on *S. aureus* cell surface were explored to explain the increased susceptibility to bacitracin. Based on our results, two potential mechanisms were proposed: disruption of the cell surface of *S. aureus* by colistin to increase entry of bacitracin into the cells and enhancement of leakage of divalent ions by colistin to enhance bacitracin antibacterial ability.

Destruction of *S. aureus* surface by colistin was supported by our results. In this study, *S. aureus* BA01611 exposed to colistin had an increased autolysis. In addition, colistin decreased positive charges on *S. aureus* cell surface. This could lead to unstable bacterial cells, as networks of metal cations could influence the rigidity and porosity of the cell wall (Silhavy et al., 2010). Furthermore, observed ion leakage from the bacterial cells after colistin exposure suggested increased *S. aureus* membrane permeability. As reviewed by Karaiskos et al. (2017), permeabilization of the bacterial membrane by colistin may allow the access of a second drug through the outer membrane into the periplasmic space and the cytoplasm, consequently improving the bactericidal activity against Gram-negative bacteria (Karaiskos et al., 2017). Electron microscopic study has also shown the cytoplasmic membrane of Gram-negative bacteria appeared to be damaged and part of the cytoplasmic material released in fibrous forms through the cracks when exposed to colistin (Koike et al., 1969). Thus, it is plausible that colistin can also modify cell structure of *S. aureus* and allow more bacitracin to get into the cytoplasm. Such a possibility needs further studies to be confirmed.

The increased susceptibility to bacitracin by colistin may also be due to the rearrangement of electric charges on cell surface, leading to the enhanced efficacy of bacitracin. As described above, *S. aureus* showed a higher negative charge of the cells and reduced cellular concentrations of Ca^2+^, Mn^2+^, Zn^2+^, and Mg^2+^ after being exposed to colistin. Bacitracin affects the biosynthesis of the peptidoglycan of bacterial cell walls by binding very tightly to the long-chain C55-isoprenol pyrophosphate and preventing its recycling (Stone and Strominger, 1973). These processes need a divalent metal ion and the antimicrobial activity of bacitracin could be stimulated by various metal ions such as Cu^2+^, Mn^2+^ or Zn^2+^ (Stone and Strominger, 1971; Ming and Epperson, 2002). In Gram-negative bacteria, the polycationic ring of colistin binds to LPS by displacement, in a competitive fashion, of the calcium (Ca^2+^) and magnesium (Mg^2+^) ions from the phosphate groups of LPS, leading to disruption of the outer membrane and loss of cellular contents and consequently killing the bacterium (Bialvaei and Samadi, 2015). We hypothesize that colistin may be capable of modulating the teichoic acid net charge by displacing the divalent cations from the phosphate groups of teichoic acids in the cell surface of *S. aureus* and thus enhancing the inhibitory effect of bacitracin on *S. aureus*.

## Conclusion

Our study showed the combination of colistin and bacitracin could be feasible for treating *S. aureus* infections. While colistin has attracted considerable interest as an antibiotic for use against multidrug-resistant Gram-negative bacteria, its potential for Gram-positive bacteria such as *S. aureus* should be further explored.

## Author Contributions

WS, VU, and XZ designed the research. WS and LW performed the research. WS, LW, and XZ analyzed the data. WS and XZ wrote the paper.

## Conflict of Interest Statement

The authors declare that the research was conducted in the absence of any commercial or financial relationships that could be construed as a potential conflict of interest.

## References

[B1] Askari E. Zarifian A. Pourmand M. Naderi-Nasab M. (2015). High-level vancomycin-resistant *Staphylococcus aureus* (VRSA) in iran: a systematic review. *J. Med. Microbiol.* 1 53–61.

[B2] Bax H. I. de Steenwinkel J. E. ten Kate M. T. van der Meijden A. Verbon A. Bakker Woudenberg I. A. (2015). Colistin as a potentiator of anti-TB drug activity against *Mycobacterium tuberculosis*. *J. Antimicrob. Chemother.* 70 2828–2837. 10.1093/jac/dkv194 26183185

[B3] Bialvaei A. Z. Samadi K. H. (2015). Colistin, mechanisms and prevalence of resistance. *Curr. Med. Res. Opin.* 31 707–721. 10.1185/03007995.2015.1018989 25697677

[B4] Biswas S. Brunel J. M. Dubus J. C. Reynaud-Gaubert M. Rolain J. M. (2012). Colistin: an update on the antibiotic of the 21st century. *Expert. Rev. Anti. Infect. Ther.* 10 917–934. 10.1586/eri.12.78 23030331

[B5] Chapnick E. K. Gradon J. D. Kreiswirth B. Lutwick L. I. Schaffer B. C. Schiano T. D. (1996). Comparative killing kinetics of methicillin-resistant *Staphylococcus aureus* by bacitracin or mupirocin. *Infect. Control. Hosp. Epidemiol.* 17 178–180. 8708360 10.1086/647270

[B6] Clinical and Laboratory Standards Institute. (2015). *Methods for Dilution Antimicrobial Susceptibility Tests for Bacteria that Grow Aerobically; Approved Standard*, 10th Edn. Wayne, PA: CLSI.

[B7] Friães A. Resina C. Manuel V. Lito L. Ramirez M. Melo-Cristino J. (2015). Epidemiological survey of the first case of vancomycin-resistant *Staphylococcus aureus* infection in europe. *Epidemiol. Infect.* 143 745–748. 10.1017/S0950268814001423 24901752 PMC9507090

[B8] Gette M. T. Marks J. G. Maloney M. E. (1992). Frequency of postoperative allergic contact dermatitis to topical antibiotics. *Arch. Dermatol.* 128 365–367. 1532297

[B9] Gordon R. J. Lowy F. D. (2008). Pathogenesis of methicillin-resistant *Staphylococcus aureus* infection. *Clin. Infect. Dis.* 46 S350–S359. 10.1086/533591 18462090 PMC2474459

[B10] Haaber J. Friberg C. McCreary M. Lin R. Cohen S. N. Ingmer H. (2015). Reversible antibiotic tolerance induced in *Staphylococcus aureus* by concurrent drug exposure. *MBio* 6:e2268–14. 10.1128/mBio.02268-14 25587013 PMC4313918

[B11] Hendley J. O. Ashe K. M. (1991). Effect of topical antimicrobial treatment on aerobic bacteria in the stratum corneum of human skin. *Antimicrob. Agents Chemother.* 35 627–631. 2069368 10.1128/aac.35.4.627 PMC245070

[B12] Hiramatsu K. L. Cui L. Kuroda M. Ito T. (2001). The emergence and evolution of methicillin-resistant *Staphylococcus aureus*. *Trends Microbiol.* 9 486–493. 11597450 10.1016/s0966-842x(01)02175-8

[B13] Johnson B. A. Anker H. Meleney F. L. (1945). Bacitracin: a new antibiotic produced by a member of the B. *Subtilis* group. *Science.* 102 376–377. 17770204 10.1126/science.102.2650.376

[B14] Karaiskos I. Souli M. Galani I. Giamarellou H. (2017). Colistin: still a lifesaver for the 21st century? *Expert. Opin. Drug Metab. Toxicol.* 13 59–71. 27573251 10.1080/17425255.2017.1230200

[B15] Koike M. Iida K. Matsuo T. (1969). Electron microscopic studies on mode of action of polymyxin. *J. Bacteriol.* 97 448–452. 4303542 10.1128/jb.97.1.448-452.1969 PMC249634

[B16] Li J. Rayner C. R. Nation R. L. Owen R. J. Spelman D. Tan K. E. (2006). Heteroresistance to colistin in multidrug-resistant *Acinetobacter baumannii*. *Antimicrob. Agents Chemother.* 50 2946–2950. 16940086 10.1128/AAC.00103-06 PMC1563544

[B17] Li L. Zhou L. Wang L. Xue H. Zhao X. (2015). Characterization of methicillin-resistant and-susceptible staphylococcal isolates from bovine milk in northwestern china. *PLoS One* 10:e0116699. 10.1371/journal.pone.0116699 25756992 PMC4355487

[B18] Limbago B. M. Kallen A. J. Zhu W. Eggers P. McDougal L. K. Albrecht V. S. (2014). Report of the 13th vancomycin-resistant *Staphylococcus aureus* isolate from the united states. *J. Clin. Microbiol.* 52 998–1002. 10.1128/JCM.02187-13 24371243 PMC3957794

[B19] Mataraci E. Dosler S. (2012). In vitro activities of antibiotics and antimicrobial cationic peptides alone and in combination against methicillin-resistant *Staphylococcus aureus* biofilms. *Antimicrob. Agents Chemother.* 56 6366–6371. 10.1128/AAC.01180-12 23070152 PMC3497160

[B20] Meehl M. Herbert S. Götz F. Cheung A. (2007). Interaction of the GraRS two-component system with the VraFG ABC transporter to support vancomycin-intermediate resistance in *Staphylococcus aureus*. *Antimicrob. Agents Chemother.* 51 2679–2689. 17502406 10.1128/AAC.00209-07 PMC1932546

[B21] Miksusanti M. Jenie B. S. L. Priosoeryanto B. P. Syarief R. Rekso G. T. (2008). Mode of action *Temu kunci* (*Kaempferia pandurata*) essential oil on E. coli K1. 1 cell determined by leakage of material cell and salt tolerance assays. *HAYATI J. Biosci.* 15 56–60. 10.4308/hjb.15.2.56

[B22] Ming L. J. Epperson J. D. (2002). Metal binding and structure–activity relationship of the metalloantibiotic peptide bacitracin. *J. Inorg. Biochem.* 91 46–58. 10.1016/S0162-0134(02)00464-6 12121761

[B23] Mun S. H. Joung D. K. Kim Y. S. Kang O. H. Kim S. B. Seo Y. S. (2013). Synergistic antibacterial effect of curcumin against methicillin-resistant *Staphylococcus aureus*. *Phytomedicine* 20 714–718. 10.1016/j.phymed.2013.02.006 23537748

[B24] Naghmouchi K. Baah J. Hober D. Jouy E. Rubrecht C. Sané F. (2013). Synergistic effect between colistin and bacteriocins in controlling gram-negative pathogens and their potential to reduce antibiotic toxicity in mammalian epithelial cells. *Antimicrob. Agents Chemother.* 57 2719–2725. 10.1128/AAC.02328-12 23571533 PMC3716138

[B25] Pankuch G. A. Lin G. Seifert H. Appelbaum P. C. (2008). Activity of meropenem with and without ciprofloxacin and colistin against *Pseudomonas aeruginosa* and *Acinetobacter baumannii*. *Antimicrob. Agents Chemother.* 52 333–336. 17967915 10.1128/AAC.00689-07 PMC2223893

[B26] Silhavy T. J. Kahne D. Walker S. (2010). The bacterial cell envelope. *Cold Spring Harb. Perspect. Biol.* 2:a000414 10.1101/cshperspect.a000414 PMC2857177 20452953

[B27] Song J. Y. Lee J. Heo J. Y. Noh J. Y. Kim W. J. Cheong H. J. (2008). Colistin and rifampicin combination in the treatment of ventilatorassociated pneumonia caused by carbapenem-resistant *Acinetobacter baumannii*. *Int. J. Antimicrob. Agents.* 32 281–284. 10.1016/j.ijantimicag.2008.04.013 18650070

[B28] Soto N. E. Vaghjimal A. Stahl-Avicolli A. Protic J. R. Lutwick L. I. Chapnick E. K. (1999). Bacitracin versus mupirocin for *Staphylococcus aureus* nasal colonization. *Infect. Control Hosp. Epidemiol.* 20 351–353. 10349956 10.1086/501633

[B29] Souli M. Rekatsina P. D. Chryssouli Z. Galani I. Giamarellou H. Kanellakopoulou K. (2009). Does the activity of the combination of imipenem and colistin in vitro exceed the problem of resistance in metallo-beta-lactamase-producing *Klebsiella pneumoniae* isolates? *Antimicrob. Agents Chemother.* 53 2133–2135. 10.1128/AAC.01271-08 19258266 PMC2681524

[B30] Spann C. T. Tutrone W. D. Weinberg J. M. Scheinfeld N. Ross B. (2009). Topical antibacterial agents for wound care: a primer. *Dermatol. Surg.* 29 620–626. 12786706 10.1046/j.1524-4725.2003.29143.x

[B31] Stone K. J. Strominger J. L. (1971). Mechanism of action of bacitracin: complexation with metal ion and C55-isoprenyl pyrophosphate. *Proc. Natl. Acad. Sci.* 68 3223–3227. 4332017 10.1073/pnas.68.12.3223 PMC389626

[B32] Stone K. J. Strominger J. L. (1973). Complex formation between bacitracin peptides and isoprenyl pyrophosphates the specificity of lipid-peptide interactions. *J. Biol. Chem.* 248 3940–3945. 4350651

[B33] Storm D. R. Rosenthal K. S. Swanson P. E. (1977). Polymyxin and related peptide antibiotics. *Annu. Rev. Biochem.* 46 723–763. 197881 10.1146/annurev.bi.46.070177.003451

[B34] Ventola C. L. (2015). The antibiotic resistance crisis Part 1: causes and threats. *P T* 40 277–283. 25859123 PMC4378521

[B35] Warren H. S. Kania S. A. Siber G. R. (1985). Binding and neutralization of bacterial lipopolysaccharide by colistin nonapeptide. *Antimicrob. Agents Chemother.* 28:107. 2412488 10.1128/aac.28.1.107 PMC176319

[B36] Werner A. H. Russell A. D. (1999). Mupirocin, fusidic acid and bacitracin: activity, action and clinical uses of three topical antibiotics. *Vet. Dermatol.* 10 225–240. 10.1046/j.1365-3164.1999.00185.x 34644919

[B37] WHO. (2014). *WHO’s first global report on antibiotic resistance reveals serious, worldwide threat to public health*. Available at: http://www.who.int/mediacentre/news/releases/2014/amr-report/en/

[B38] Wu Z. Li F. Liu D. Xue H. Zhao X. (2015). ). Novel type XII staphylococcal cassette chromosome mec harboring a new cassette chromosome recombinase, CcrC2. *Antimicrob*. *Agents Chemother.* 59 7597–7601. 10.1128/AAC.01692-15 26416872 PMC4649182

[B39] You Y. O. Choi N. Y. Kang S. Y. Kim K. J. (2013). Antibacterial activity of rhus javanica against methicillin-resistant *staphylococcus aureus*. *Evid. Based Complement. Altern. Med.* 2:549207. 10.1155/2013/549207 24223060 PMC3816054

[B40] Yu Z. Qin W. Lin J. Fang S. Qiu J. (2015). Antibacterial mechanisms of polymyxin and bacterial resistance. *Biomed. Res. Int.* 2015:679109. 10.1155/2015/679109 25664322 PMC4312571

